# Tropomyosin Period 3 Is Essential for Enhancement of Isometric Tension in Thin Filament-Reconstituted Bovine Myocardium

**DOI:** 10.1155/2009/380967

**Published:** 2009-10-13

**Authors:** Masataka Kawai, Xiaoying Lu, Sarah E. Hitchcock-DeGregori, Kristen J. Stanton, Michael W. Wandling

**Affiliations:** ^1^Department of Anatomy and Cell Biology, The University of Iowa, Iowa City, IA 52242, USA; ^2^Department of Neuroscience and Cell Biology, Robert Wood Johnson Medical School, Piscataway, NJ 08854, USA

## Abstract

Tropomyosin (Tm) consists of 7 quasiequivalent repeats known as “periods,” and its specific function may be associated with these periods. To test the hypothesis that either period 2 or 3 promotes force generation by inducing a positive allosteric effect on actin, we reconstituted the thin filament with mutant Tm in which either period 2 (Δ2Tm) or period 3 (Δ3Tm) was deleted. We then studied: isometric tension, stiffness, 6 kinetic constants, and the pCa-tension relationship. N-terminal acetylation of Tm did not cause any differences. The isometric tension in Δ2Tm remained unchanged, and was reduced to ∼60% in Δ3Tm. Although the kinetic constants underwent small changes, the occupancy of strongly attached cross-bridges was not much different. The Hill factor (cooperativity) did not differ significantly between Δ2Tm (1.79 ± 0.19) and the control (1.73 ± 0.21), or Δ3Tm (1.35 ± 0.22) and the control. In contrast, pCa_50_ decreased slightly in Δ2Tm (5.11 ± 0.07), and increased significantly in Δ3Tm (5.57 ± 0.09) compared to the control (5.28 ± 0.04). These results demonstrate that, when ions are present at physiological concentrations in the muscle fiber system, period 3 (but not period 2) is essential for the positive allosteric effect that enhances the interaction between actin and myosin, and increases isometric force of each cross-bridge.

## 1. Introduction

The tropomyosin (Tm) gene is the product of gene duplication [1] and its sequence reflects the presence of a 7-fold repeat that has been postulated to relate to the binding sites for the 7 actin monomers along the length of a Tm molecule [1–3]. This molecule is a coiled-coil dimeric protein, and together with the troponin complex (Tn), it regulates thin-filament activation in striated muscles. This event is initiated by Ca^2+^ binding to TnC, and enhanced by myosin binding to actin. The reactions are cooperative and allosteric, as shown in solution studies [4–6], in vitro motility assays [7–10], and in muscle fiber studies [11–14]. Analyses of the mechanism that underlies thin-filament activation have indicated a role for the 7 internal quasiequivalent repeats, called “periods,” in this activation process [15–19] as well as a role for the head-to-tail association of adjacent Tm molecules [20]. 

Further analysis of mutants with deletions of one or more of the periodic repeats–by solution studies, in vitro motility assays, and muscle-fiber analysis–has revealed the importance of the internal periods for allosteric regulation of actin-filament activation. Specifically, an actomyosin ATPase study and in vitro motility assays have shown that the calcium ion poorly activates actin filaments that are reconstituted with Tn plus a Tm harboring a period 3 deletion (either individually or in combination with the deletion of another period) [16, 19]. Later reports showed that the same deletions reduce the activity in the in vitro motility assay [10, 17] as well as the isometric tension [13], by ~50%.

Experiments using skinned fibers are important, because force can be measured in these preparations and the measurements can be performed in solutions that are at physiological ionic strength. Extraction of the thin filaments from strips of bovine myocardium, followed by structural and stoichiometric reconstitution of the muscle with natural or recombinant proteins, generates a suitable model for such experiments [11, 12, 21–23]. The advantage of this system is that it makes possible the measurement of isometric tension and its kinetic constants in the context of a mutant protein. The use of solutions at physiological ionic strength is important because the electrostatic interaction becomes stronger at lower ionic strength, obliterating the significance of the hydrophobic interaction. 

**Figure 1:**
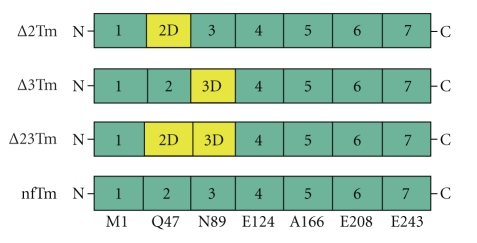
Tropomyosins used for reconstitution. The structure of *E*. *coli*-synthesized Tm used for our study, highlighting the 7 quasirepeat regions. The N-terminal amino acid residue for each region is shown underneath nfTm. Yellow color represents the region(s) deleted in the respective mutants. ∆23Tm was previously studied [13, 14] and is included here for the sake of comparison. nfTm is the full-length protein and was used as a control. The N-terminus of ∆23Tm has the Ala-Ser substitution, whereas the N-termini of ∆2Tm, ∆3Tm, and nfTm do not have this substitution.

Investigating the consequences of the deletion of individual periods by carrying out solution studies suggested that the regulatory function of Tm is associated with certain periods [19]. Additional investigations using in vitro motility assays [10, 17] and thin filament-reconstituted muscle fibers [13, 14] have indicated that a truncated Tm mutant, ∆23Tm (Figure 1), has a negative effect on the allosteric changes normally involved in the actomyosin interaction. Specifically, the isometric tension in a myocardial model reconstituted with ∆23Tm was only ~40% of that in a model reconstituted with the control protein [13]. The number of force-generating cross-bridges was not reduced, indicating that the tension per cross-bridge was reduced. The temperature dependence of the tension change under normal conditions indicated that hydrophobic interactions are involved in force generation (reviewed by [24]). This interaction was significantly reduced when ∆23Tm was applied [14]. In the present work, we build on these findings by showing that the negative allosteric effects of the ∆23Tm mutant are attributable to a lack of period 3, that is, residues 89–123 of Tm. A preliminary account of the present results was presented at a Biophysical Society Meeting [25]. 

## 2. Methods

### 2.1. Thin-Filament Extraction and Reconstitution in Myocardium

The technique of thin-filament extraction and reconstitution was performed as described in [11, 13, 21] and reviewed recently in [23]. In brief, strips of bovine myocardium (length ~3 mm, diameter 90–110 *μ*m) are mounted between a length driver and a force transducer, and treated in relaxing solution that contained 1% Triton X100 at 0°C for 20 minutes. The thin filament is then removed by treatment with gelsolin (previously called brevin [26]) for 45–120 min, which results in fibers bare of thin filament, except in the immediate vicinity of the Z-line [21]. Connectin (titin), which anchors the thick filament to the Z-line, is not disturbed by this technique. The actin filament is then reconstituted by adding exogenous G-actin under polymerizing conditions. Thin-filament reconstitution is completed by reconstituting both Tm and Tn at the same time. Throughout the extraction-reconstruction procedure, 2,3-butanedione 2-monoxime (BDM) is used to inhibit the actomyosin interaction [27–29]. Modifications in this study are the use of (1) 56 mM, rather than 80 mM, KI during actin polymerization, and (2) the incorporation of a 5th solution change [11]. Consequently, the duration of the actin polymerization procedure was 35 minutes instead of 28 minutes. 

The Tm molecules used for reconstitution included nfTm, ∆2Tm, ∆3Tm, and acetyl Tm (Figure 1). The recombinant Tms are encoded by the chicken *α*-Tm gene (*TPM1*). The sequence of nfTm is that of striated *α*-Tm (the same sequence for both skeletal and cardiac muscles), ∆2Tm is the same but lacks the period 2-encoding sequence (residues 47–88) [30], and ∆3Tm is the same but lacks the period 3-encoding region (residues 89–123) [15]. nf stands for nonfusion peptide, and it does not have N-terminal acetylation or substitution with Ala-Ser N-terminal fusion peptide. The bovine and chicken striated muscle *α*-Tms are 95% identical in sequence. There are 9 amino acid differences located throughout the sequence. The differences are generally conservative in nature: D to E, A to S, S to T. Extensive analysis has shown that there are minor differences in affinity and stability between the isoforms, but no qualitative differences were reported. The previously published deletion mutant, in which periods 2 and 3 were deleted, was made in both chicken and rat, and had very similar regulatory properties [15, 16], even when used with skeletal chicken versus cardiac bovine Tn. Therefore, the species difference should be of minor consequence. The recombinant proteins are chicken, because the original mutants were prepared in a chicken cDNA. nfTm, ∆2Tm and ∆3Tm were expressed in *E . coli* and purified as described [19], and thus their N-terminal Met residues are unacetylated. Acetyl Tm was purified from bovine myocardium; most of the data on acetyl Tm were reported previously [13] and are incorporated in both the Tables and Figure 3(c) for the sake of comparison. 

Actin was purified from acetone powder, a gift from Dr. Shin'ichi Ishiwata of Waseda University in Tokyo and had been isolated from New Zealand white rabbit's fast twitch (white) muscles. Tn was a gift from Dr. Larry S. Tobacman of The University of Illinois at Chicago and had been purified from bovine heart as reported in [31]. 

Isometric force was measured at the completion of each step of the extraction/reconstitution procedure, following brief stimulation (application of the standard activating solution at 25°C) and immediate relaxation (application of the relaxing solution at 0°C). The data on force, stiffness and rigor stiffness that were subsequently gathered, were normalized to the isometric force (*T*_ac_) of the preparation when the actin filament was reconstituted. In this way, the scatter of the data due to inaccurate estimation of the fiber cross-sectional area and the possible variable length of the reconstituted thin filament can be minimized.

### 2.2. Mechanical Studies

The reconstituted preparations were studied with 4 series of solutions that changed (1) pCa (= –log[Ca^2+^]), (2) pS (= –log[MgATP^2−^]), (3) [MgATP^2−^], and (4) [Pi^1.5−^]. (2) was performed in the absence of Ca^2+^; (3) and (4) were performed in the presence of saturating Ca^2+^. Towards the end, the fibers were activated by the standard activating solution, then thereafter rigor was induced with 2 solution changes. All activations including that of rigor were performed at 25°C, except for those that were part of the temperature study. All relaxation was performed at 0°C. At the termination of each mechanical experiment, the preparation was removed from the experimental apparatus, dissolved in sample dilution buffer, and pooled for SDS-PAGE analysis. 

For statistical analysis, a paired *t*-test was performed using ANOVA and assuming unequal variances, and the results were compared to those of the nfTm-reconstituted group (control).

### 2.3. pCa-Tension and pCa-Stiffness Studies

The reconstituted fibers were first studied with 13 different Ca^2+^ concentrations (pCa: 7.0, 6.4, 6.2, 6.0, 5.8, 5.6, 5.4, 5.2, 5.0, 4.66, 4.3, 4.0, 3.5) at the fixed [MgATP] (5 mM) and [Pi] (8 mM). The tension data were then fitted to the Hill equation:

(1) $$T=\frac{T_{m}}{1+{\left(\text{C}{\text{a}}_{50}/\left[\text{C}{\text{a}}^{2+}\right]\right)}^{h}}+T_{b},$$

where *T* is isometric tension, *T*_*m*_ is the maximum tension at saturating Ca^2+^, and *T*_*b*_ is the baseline tension. Ca_50_ is the dissociation constant of Ca^2+^, which represents [Ca^2+^] at half saturation. pCa_50_ (= –log Ca_50_) is called the Ca^2+^ sensitivity. *h* is the Hill factor and represents the cooperativity of activation. The data are plotted after subtraction of *T*_*b*_ and normalization to *T*_*m*_. The stiffness data were analyzed with the equation similar to (1).

### 2.4. pS-Stiffness and pS-Tension Studies

The effect of pS (= –log[S], where S = MgATP) on stiffness and tension was studied at every 0.2 pS unit where stiffness and tension change quickly, else, studied less frequently. This experiment was performed in the absence of Ca^2+^ and in the presence of 6 mM K_2_EGTA. Stiffness was measured at 100 Hz. The data were fitted to (2), which is similar (but not identical) to (1):

(2) $$Y_{100}=\frac{Y_{m100}}{1+{\left(S/S_{50}\right)}^{g}}+Y_{b100},$$

where *S*_50_ is the dissociation constant of MgATP, which is the MgATP concentration at half relaxation, *g* represents the cooperativity of relaxation, *Y*_100_ is stiffness measured at 100 Hz, *Y*_*m*100_ is the maximum stiffness in the absence of MgATP, and *Y*_*b*100_ is the baseline stiffness at high MgATP concentration. The data were plotted after the subtraction of the baseline stiffness (*Y*_*b*100_) and normalization to *Y*_*m*100_. The tension data are fitted to an equation similar to (2), where only the data to the right of the peak are used.

### 2.5. Sinusoidal Analysis

At each activation, the computer was triggered and sinusoidal length changes were applied at a small amplitude (0.125%) and 18 different frequencies in the range 0.13 and 100 Hz. Isometric force and force transients were recorded using the same computer, signal averaged, and the complex modulus *Y*(*f*) was calculated. *Y*(*f*) is a frequency (*f*) response function, which is the ratio of the stress change to the strain change expressed in the frequency domain. *Y*(*f*) was displayed on the computer screen immediately after the measurements were taken; it was further fitted to (3), which incorporates three exponential processes, A, B, and C [32]:

(3) $$Y\left(f\right)=H+\frac{Afi}{a+fi}-\frac{Bfi}{b+fi}+\frac{Cfi}{c+fi},$$

where $i=\sqrt{-1}$; 2*πa*, 2*πb*, and 2*πc*, respectively correspond to the apparent rate constants of processes A, B, and C; *A*, *B*, and *C* are their respective magnitudes (amplitudes). In cardiac muscle fibers, process A is absent (*A* = 0) at ≤25°C [11, 33, 34], but it plays a role at temperatures ≥30°C [14]. From (3), we have

(4) $$Y_{\infty }\equiv Y\left(\infty \right)=H+A-B+C$$

*Y*_*∞*_ is the complex modulus extrapolated to the infinite frequency. *Y*_*∞*_ corresponds to phase 1, process C corresponds to phase 2, process B to phase 3, and process A to phase 4 of step analysis [32].

### 2.6. The Effect of MgATP on the Apparent Rate Constant 2*π*c

**Scheme 1:**
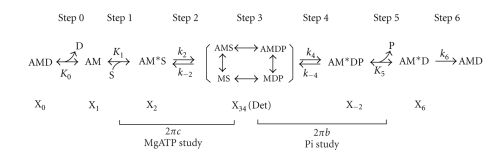
Six-state cross-bridge model, where A = actin, M = myosin, D = MgADP, S = MgATP, and P = Pi = phosphate.

The preparations were studied with 7 different MgATP concentrations (0.05, 0.1, 0.2, 0.5, 1, 5, 10 mM) at the fixed [Pi] (8 mM) in the presence of saturating Ca^2+^. To deduce the rate and association constants of steps 1 and 2 (Scheme 1), the effect of [MgATP] on the apparent rate constant 2*πc* was fitted to the following [35]:

(5) $$2\pi c=\frac{K_{1}S}{1+K_{1}S}k_{2}+k_{-2},$$

where *S* = [MgATP], *K*_1_ is the association constant of MgATP to the myosin head, *k*_2_ is the rate constant of cross-bridge detachment step, and *k*_−2_ is that of its reversal step.

### 2.7. The Effect of Pi on the Apparent Rate Constant 2*π*b

The effect of phosphate (Pi) was studied at 6 different Pi concentrations (0, 2, 4, 8, 16, 32 mM: added concentrations) in the presence of the fixed [MgATP] (5 mM) and saturating Ca^2+^. To deduce the rate and association constants of steps 4 and 5 (Scheme 1), the effect of [Pi] on the apparent rate constant 2*πb* was fitted to the following [35]:

(6) $$2\pi b=\sigma k_{4}+\frac{K_{5}P}{1+K_{5}P}k_{-4},$$

where

(7) $$\sigma =\frac{K_{1}SK_{2}}{1+K_{1}S\left(1+K_{2}\right)},$$

and *P* = [*P*_*i*_]. *k*_4_ is the rate constant of the step 4 (force generation step), *k*_−4_ is that of its reversal step, and *K*_5_ is the association constant of the Pi molecule to the myosin head. *σ* of (7) was calculated from *K*_1_ and *K*_2_ obtained from the MgATP study (5) with *S* = 5 mM.

### 2.8. Biochemical Analysis of Extracted and Reconstituted Myocardium

To prepare samples for SDS-PAGE, which requires a large quantity of muscle tissue relative to mechanical studies, an additional group of 10–15 fibers/mutant was suspended between two stainless steel (ss) wires (diameter 500 *μ*m, length 15 mm, 4 mm apart) after being glued to them with nail polish (Figure 10). A 3rd ss wire was placed in the center to prevent fibers from breaking when they cross the meniscus (the fibers were not glued to this central ss wire). These muscle fibers were placed into Eppendorf tubes and subjected to the same extraction and reconstitution procedures as the fibers that were used for mechanical analysis. Each solution (200 *μ*l) was added at 0°C. Once reconstituted, the fibers were dissolved in the sample dilution buffer together with fibers used for mechanical analysis and were then heated to 100°C for 5 minutes. SDS-PAGE, using an 8–16% acrylamide gradient gel (with a 4% stacking gel: Bio-Rad, Cat. No. 161–1223), was then performed. The samples were electrophoresed at 45 mA (~200 V) for about 40 min, and stained with Coomassie brilliant blue R250 as reported [36]. The gels were photographed using a high-resolution digital camera, and the intensity of each band was measured using the UN-SCAN-IT (version 5.1) program.

### 2.9. Solutions

The Na-skinning solution contained (mM) 10 K_2_EGTA, 2 Na_2_MgATP, 5 Na_2_K_2_ATP, 122 NaAc (Ac *=* acetate), 10 MOPS, and 30 BDM. The K-skinning solution contained 10 K_2_EGTA, 2 Na_2_MgATP, 5 Na_2_K_2_ATP, 122 KAc, 10 MOPS, and 30 BDM. The storage solution contained 10 K_2_EGTA, 2 Na_2_MgATP, 5 Na_2_K_2_ATP, 122 KAc, 10 MOPS, 30 BDM, and 6 M glycerol. The relaxing solution contained 6 EGTA, 2.2 Na_2_MgATP, 5 Na_2_K_2_ATP, 8 K_1.5_Pi, 41 NaProp, 75 KProp, 10 MOPS, and 40 BDM. The thin filament extraction solution contained 2 CaEGTA, 2.2 Na_2_K_2_ATP, 121 KCl, 4.25 MgCl_2_, 2 leupeptin, 2 diisopropyl fluorophosphate (DFP), 40 BDM, 20 MOPS, and ~0.3 mg/ml purified **gelsolin** from bovine plasma. The actin filament reconstitution solution contained (mM) 4 EGTA, 4 Na_2_MgATP, 32 KCl, 56 KI, 40 BDM, 20 K_1.5_Pi, and 1 mg/ml purified **G-actin** from rabbit back muscles. The Tm/Tn reconstitution solution contained ~0.6 mg/ml  **Tm** and ~0.6 mg/ml bovine cardiac **Tn** in the relaxing solution. The standard activating solution contained 6 CaEGTA, 5.8 Na_2_MgATP, 1.36 Na_2_K_2_ATP, 15 creatine phosphate (Na_2_CP), 4 KH_2_PO_4_, 4 K_2_HPO_4_, 1 NaProp, 73 KProp, 10 NaN_3_, 10 MOPS, and 320 U/ml CK (pCa 4.66, total Na 55 mM, ionic strength 200 mM). Any other activating solution was a variant of the standard activating solution, in which ionic strength was maintained at 200 mM and the total [Na] at 55 mM. The rigor solution contained 55 NaProp, 4 KH_2_PO_4_, 4 K_2_HPO_4_, 122 KProp, and 10 MOPS. The pH of each solution was adjusted to 7.00. The concentrations of multivalent ionic species were calculated using our computer program ME, based on the following apparent association constants (log values at pH 7.00): CaEGTA = 6.28, MgEGTA = 1.61, CaATP = 3.70, MgATP = 4, CaCP = 1.15, and MgCP = 1.30.

## 3. Results

### 3.1. Mechanical Studies of Thin Filament-Reconstituted Fibers

**Figure 2:**
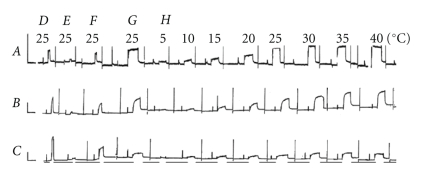
Slow pen trace of isometric tension in the standard activating solution (pCa 4.66, 5 mM MgATP, 8 mM Pi, ionic strength 200 mM, 15 mM CP, 320 units/ml CK, pH 7.00) at different temperatures (indicated). Experiments were performed in thin filament-reconstituted cardiac muscle fibers, in which nfTm (*A*), ∆2Tm (*B*), and ∆3Tm (*C*) were added back, together with bovine cardiac Tn. *D*, activation of native fibers; *E*, activation of thin filament-extracted fibers after gelsolin treatment for 50–90 minutes; *F*, activation of actin filament-reconstituted fibers; and *G*, activation of Tm- and Tn-reconstituted fibers. *H*, activation at eight different temperatures, as indicated. Between *D*, *E*, *F*, and *G*, the pen recorder was stopped so that extraction and reconstitution could be performed (at 0°C). Before each activation, the muscle fibers were washed in the standard activating solution (at 0°C), which did not induce tension. Tension was induced by switching the fibers to a bath of the same solution at the higher temperature. The fibers were relaxed in the Rx solution that contained 40 mM BDM at 0°C. Horizontal bars below the pen trace in *C* indicate that the temperature was 0°C during relaxation. The active tension in *F* develops as the result of withdrawal of BDM, and the temperature rises to 25°C. The active tension is relaxed as the result of the addition of 40 mM BDM, and the temperature drops to 0°C. Calibrations are 1 min (abscissa) and 10 kPa (ordinate).

To compare fiber mechanics between mutant and control Tms, and to establish the structure-function relationship for periods 2 and 3 of this protein, we dissolved bovine myocardium thin filaments with gelsolin, and then reconstituted them with component proteins as described in [11, 21]. The effects of fiber reconstitution on isometric tension are illustrated in Figure 2. Panel D shows control activation of the native myocardium with the standard activating solution, and E shows the consequences of gelsolin treatment (for 60–120 min) for the same activation. In Figure 2E tension was not generated because the thin filament had been removed. Figure 2F shows that, after actin filament reconstitution, isometric tension was restored to 50–70% of that in the control [11]. Figure 2G shows the consequences of thin-filament reconstitution with nfTm (Figure 2A), ∆2Tm (Figure 2B), and ∆3Tm (Figure 2C). In the cases of reconstitution with nfTm and ∆2Tm, tension was greater than that attributable to actin-filament tension (Figure 2F), and comparable to that generated in response to initial activation in Figure 2D. However, in the case of reconstitution with ∆3Tm (Figure 2CG), tension was significantly lower than that of actin-filament reconstituted preparations (Figure 2CF). 

### 3.2. Resting Tension and Stiffness, and the Ability to Turn Off the Actomyosin Interaction at pCa 7

It is possible that the actomyosin interaction may not be completely turned off in some of the mutant Tms used for reconstitution. For this reason, tension at pCa7.0 was measured at 25°C as the incremental tension from the relaxed level that exists at 0°C in the relaxing solution that contains 40 mM BDM. All the measured parameters were normalized to *T*_ac_, averaged, and listed in Table 1 for three muscle models. The results from acetyl Tm are also included. Similarly, stiffness values at relaxation (0°C) and at pCa 7 (25°C) are listed in Table 1. As this table demonstrates, all the parameters measured are not any different from those of the nfTm control, indicating that the actomyosin interactions were indeed turned off at pH 7 with any of the Tms used. 

### 3.3. pCa-Tension and pCa-Stiffness Studies

**Figure 3:**
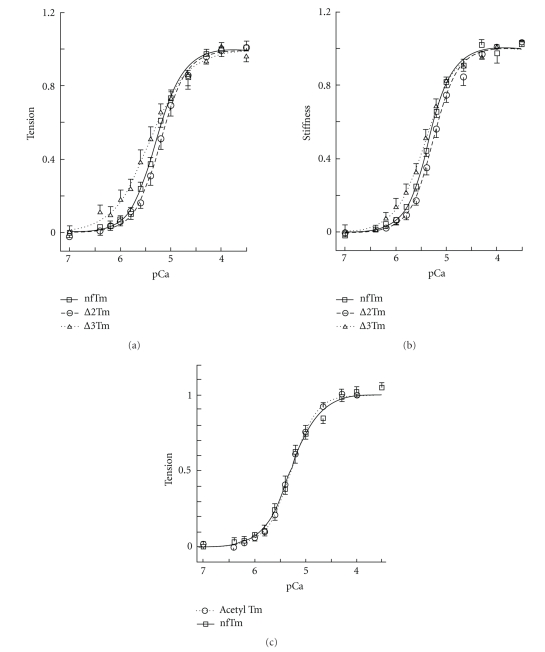
pCa-tension curves. (a) pCa-tension plot comparing ∆2Tm-, ∆3Tm-, and nfTm-reconstituted muscle fibers. The tension data were fitted to (1), and the continuous lines are drawn based on best-fit parameters (Table 2). Error bars represent SEM. *N* = 14 for nfTm, *N* = 15 for ∆2Tm and ∆3Tm, and *N* = 9 for acetyl Tm. For acetyl Tm, the fitted parameters were reported in Lu et al. [13]. (*b*) pCa-stiffness plot, fitted to an equation similar to (1). (*c*) pCa-tension plot comparing nfTm and acetyl Tm.

Whether the cooperative activation mechanism and the Ca^2+^ sensitivity of the myocardium are altered by the Tm mutants was determined by studying tension and stiffness of the thin filament-reconstituted fibers as functions of pCa. The results obtained for filaments reconstituted with nfTm, ∆2Tm, and ∆3Tm are shown in Figure 3(a) (tension) and in Figure 3(b) (stiffness). The data were fitted to (1) and the results are summarized in Table 2. Maximal tension (*T*_*m*_) at saturating Ca^2+^ and stiffness (*Y*_*∞*_) are entered in Table 4. This table demonstrates that *T*_*m*_ hardly differs between the nfTm and ∆2Tm muscle models, but the *T*_*m*_ of the ∆3Tm muscle model is only 60 ± 12% that of the nfTm model. Hence we conclude that isometric tension is significantly reduced in this muscle model. Table 2 also demonstrates that pCa_50_ is slightly decreased (by 0.17 ± 0.08 units) in the case of ∆2Tm but substantially increased (by 0.23 ± 0.09 units) in ∆3Tm. Also, cooperativity (*h*) does not differ significantly between the nfTm and ∆2Tm models, but is slightly reduced in the ∆3Tm model. A similar trend can be seen for stiffness (*Y*_*∞*_) (Table 2). Thus, the large difference in maximal tension and stiffness of the ∆3Tm mutant cannot be explained by a difference in the Ca^2+^ sensitivity. 

Because *E . coli*-synthesized Tm lacks acetylation at the N-terminal Met, and this modification is thought to be important for the head-to-tail association of the Tm molecule [37, 38], we compared the pCa-tension plots for nfTm and acetyl Tm purified from bovine myocardium (fitted parameters were reported previously [13]). The results are plotted in Figure 3(c) and are compared in Table 2. They demonstrate that there is hardly any difference in the pCa-tension plot between nfTm and acetyl Tm: ∆pCa_50_ was 0.12 ± 0.09, and ∆*h* was 0.01 ± 0.31. Therefore, we conclude that after unacetylated Tm is incorporated in the thin filament, it behaves as the normal acetylated Tm does. We further conclude that the difference in Ca^2+^ sensitivity or cooperativity associated with thin-filament activation in the presence of ∆3Tm is not a consequence of the lack of acetylation of the N-terminal Met.

### 3.4. pS-Stiffness and pS-Tension Studies

**Figure 4:**
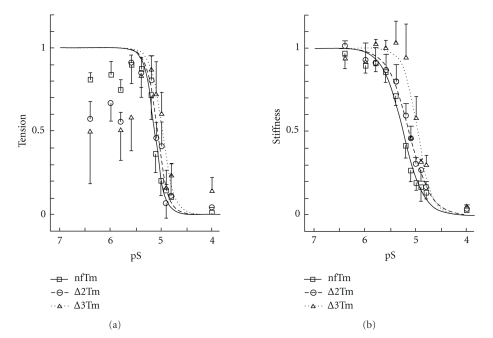
pS-tension curves. pS-tension plot (*S *= [MgATP^2−^]) in (a) and pS-stiffness plot in (b) comparing nfTm, ∆2Tm, and ∆3Tm. The stiffness data were fitted to (2), and continuous lines are drawn based on best-fit parameters. The tension data in (a) are fitted to an equation similar to (2), where only the data to the right of the peak are used for fitting. The experiments were carried out at 8 mM Pi, and in the absence of Ca^2+^. Error bars represent SEM. *N* = 5 for nfTm and ∆2Tm, and *N* = 4 for ∆3Tm.

To examine whether the cooperativity with respect to relaxation from the rigor condition is altered when mutant Tms are present, both Ca^2+^ and MgATP^2−^ were deleted from the standard activating solution to produce fibers in the “high-rigor state” [39] with large tension. In the absence of Ca^2+^, MgATP^2−^ was added to the rigor preparation incrementally, and both tension and stiffness at 100 Hz were studied as functions of pS. The results are shown in Figure 4(a) (pS-tension plot) and in Figure 4(b) (pS-stiffness plot), which compare the effects of nfTm, ∆2Tm, and ∆3Tm. As shown in Figure 4(a), increasing [MgATP] led to increased tension, which peaked at around pS = 5.4 ~ 5.6, and declined quickly over higher MgATP concentrations [39, 40]. The increase in tension was due to rigor activation of the thin filament (owing to thin-filament activation by attached cross-bridges [41]) and increased availability of MgATP to generate higher tension [39]. The stiffness (Figure 4(b)) stayed about the same in the pS range between 6.5–5.6; this indicates that the maximum number of strongly attached cross-bridges has been attained and does not change appreciably in the pS range between 6.5–5.6. The tension and stiffness then declined towards the higher MgATP concentrations, as had been demonstrated earlier using crayfish walking-leg muscle fibers [39, 40]. The decrease in tension and stiffness was due to an increase in [MgATP], which results in the detachment of myosin cross-bridges. Because this detachment disrupts cooperative activation, the relaxation was abrupt, as shown in Figure 4. The stiffness data were fitted to (2), and the results are summarized in Table 3. The ATP sensitivity (pS_50_) did not differ appreciably between the nfTm (5.12 ± 0.05) and ∆2Tm muscle models (5.00 ± 0.08) but was significantly decreased, by 0.17 ± 0.08 units, in the ∆3Tm model (4.95 ± 0.06); this indicates that a higher [MgATP] is necessary to effect relaxation in the ∆3Tm muscle model. Because of the scatter of the data, we could not tell if the cooperativity of relaxation (*g*) was any different among three muscle models (Table 3). However, its average values range from 5.9–7.8, indicating a high level of cooperativity. If the cooperativity were absent, this number would have been 1 (one). Qualitatively similar results were obtained based on stiffness, although the cooperativity was 2.0-3.0 and somewhat lower than that of tension (Table 3). 

### 3.5. Sinusoidal Analysis and the Apparent Rate Constants

**Figure 5:**
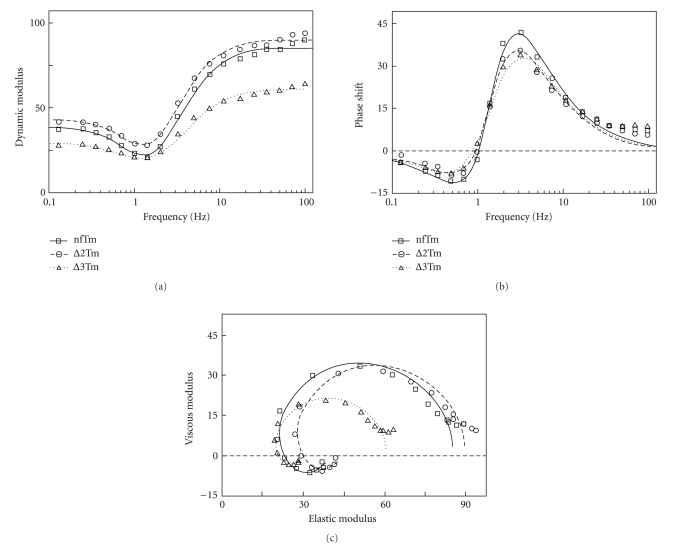
Frequency plots of complex moduli *Y*(*f*). *Y*(*f*) values during standard activation at 25°C are plotted as (a) dynamic modulus (= |*Y*(*f*)|) versus frequency, (b) phase shift (= arg*Y*(*f*)) versus frequency, and (c) viscous modulus (= Real  *Y*(*f*)) versus elastic modulus (= Imag  *Y*(*f*)) (Nyquist plot) with the frequency as a variable. Muscle models from three different Tms are compared. The averaged data of 15 (nfTm), 17 (∆2Tm), and 18 (∆3Tm) preparations are shown. Frequencies tested were 0.13, 0.25, 0.35, 0.5, 0.7, 1, 1.4, 2, 3.1, 5, 7, 11, 17, 25, 35, 50, 70, and 100 Hz (clockwise in (c)). The data are fitted to (3). The continuous curves represent represent calculated values from (3) with best-fit parameters. The units of all moduli are *T*_ac_, phase shift is expressed as degree, and frequency in Hz. The resting modulus was not subtracted.

To characterize the cross-bridge kinetics in the thin-filament reconstituted muscle fiber system, we performed sinusoidal length oscillations during standard activation, and followed tension transients as reported [32]. We prefer this method over step analysis because of its higher resolving power. The complex modulus data [*Y*(*f*)] resulting from the application of this methodology to the three muscle models are plotted in Figure 5, where each point represents the frequency of oscillation. The complex modulus data all show a typical cardiac response [33, 34]: the dynamic modulus (Figure 5(a)) is *v*-shaped, with the minimal value occurring at ~1.4 Hz (= *f*_min_), and it increases to a plateau both in the low-frequency and high-frequency ranges. The average value of 2*πf*_min_ is entered in Table 4. The phase shift (Figure 5(b)) is *s*-shaped; its minimum occurs at ~0.5 Hz and its maximum at ~3 Hz. The Nyquist plot (Figure 5(c)) for the cardiac preparations is in the form of two semicircles; its low-frequency component is in the 4th quadrant, whereas its high-frequency component is in the 1st. The general shape of the plots does not differ significantly among the three muscle models, except that in ∆3Tm the amplitude of the dynamic modulus is about 66% that of others (Figure 5(a)). Consequently, the diameter of the semicircles in the ∆3Tm muscle model is about 66% that of the other two models (Figure 5(c)). The highest frequency used (100 Hz; time resolution 1.6 milliseconds) is adequate to characterize complex modulus in cardiac muscle fibers, because both dynamic modulus (Figure 5(a)) and phase shift (Figure 5(b)) approach steady values towards 100 Hz, the exponential process B centers at around 1 Hz (= *b*), and exponential process C centers at around 5 Hz (= *c*). 

The calculated rate constants and associated parameters are summarized in Table 4, in which it can be seen that both apparent rate constants (2*πb*, 2*πc*) differ very little between the nfTm and ∆2Tm models. However, they are significantly different in the ∆3Tm model: 2*πb* is 1.4× smaller, and 2*πc* is 1.2× larger, than their counterparts in the nfTm model. These facts imply that the cross-bridge kinetics of the nfTm and ∆2Tm models are similar, but that those of the ∆3Tm model are significantly different. The magnitudes *B*, *C*, and *H* do not differ significantly between the nfTm and ∆2Tm models but are significantly lower (by 33–66%) in the ∆3Tm model. In all four muscle models tested, however, *f*_min_ is similar and falls between the characteristic frequencies *b* and *c* defined in (3).

### 3.6. Study of Elementary Steps of the Cross-Bridge Cycle

**Figure 6:**
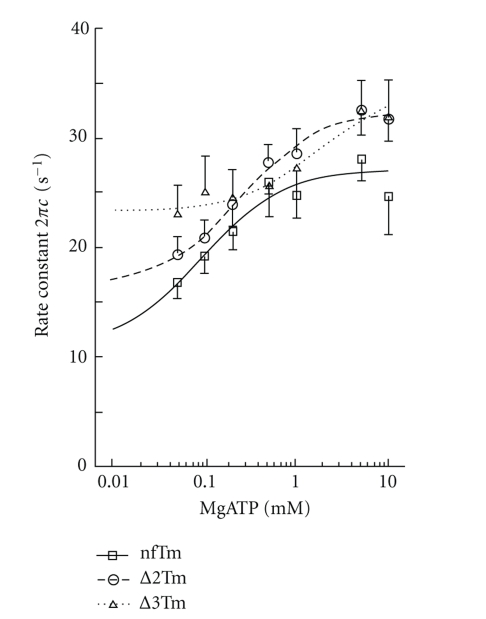
The effect of MgATP on the apparent rate constant 2*π*c. The data were fitted to (5). The Pi concentration was kept at 8 mM. The continuous lines are based on (5) with best-fit parameters.

**Figure 7:**
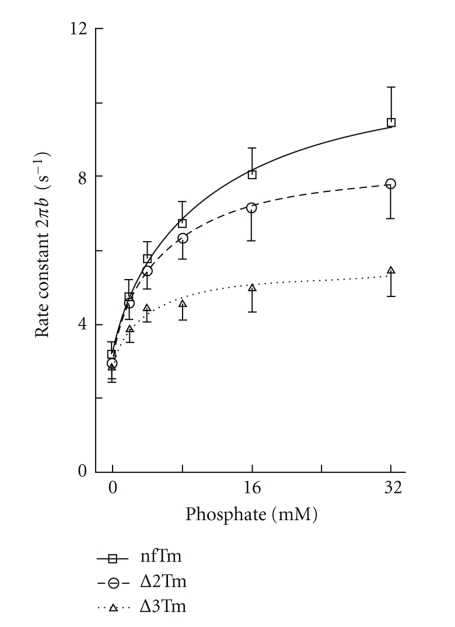
The effect of Pi on the apparent rate constant 2*π*b. To deduce the rate and association constants of steps 4 and 5, the results of Figure 7 were fitted to (6). The MgATP concentration was kept at 5 mM. The continuous lines are based on (6) with best-fit parameters. *σ* of (7) was calculated based on the *K*_1_ and *K*_2_ obtained from the MgATP study (Table 5) and at *S* = 5 mM.

To compare each elementary step of the cross-bridge cycle among the three muscle models, we studied the effects of MgATP and Pi on the apparent rate constants 2*πb* and 2*πc* in the presence of saturating Ca^2+^. The results for the MgATP study, in which [MgATP] was changed gradually from 0.05 mM to 10 mM while [Pi] was kept at 8 mM, are plotted in Figure 6; those for the Pi study, in which added [Pi] was changed gradually from 0 mM to 32 mM while [MgATP] was kept at 5 mM, are plotted in Figure 7. Results were fitted to a 6-state cross-bridge scheme (Scheme 1) [33, 35, 42]. Together, the rate and association constants of the elementary steps as defined in Scheme 1 are referred to as “kinetic constants.” These are summarized in Table 5, in which it can be seen that the kinetic constants for the nfTm and Δ2Tm models differ minimally, if at all. The largest effects are the 2.3× changes in *k*_−4_ and *K*_5_, which represent the reversal of the force-generation step and the association constant of Pi, respectively. Some kinetic constants differ significantly between the nfTm and ∆3Tm models, and the largest consequence of this is a 5.2× change in *K*_5_.

### 3.7. Cross-Bridge Distribution

**Figure 8:**
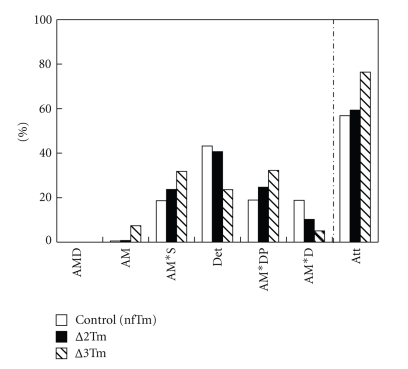
Cross-bridge distribution among 6 states during full Ca^2+^ activation. See Scheme 1 and its legends for abbreviations of the cross-bridge states. Det is the sum of all detached states (MS and MDP) and weakly attached states (AMS and AMDP). The distribution was calculated based on *K*_1_, *K*_2_, *K*_4_, *K*_5_ (Table 2), [MgATP] = 5 mM, [Pi] = 8 mM, and (7)–(13) of [43].

There are two possible reasons for the reduced isometric tension in the ∆3Tm model with respect to that in the nfTm and ∆2Tm models. (1) Fewer force-generating cross-bridges are formed in the ∆3Tm model, and (2) the force generated by each cross-bridge is less in the ∆3Tm model than in the nfTm and ∆2Tm models. To determine which of these scenarios is correct, we calculated the distribution of cross-bridges in each state according to (7)–(13) in [43]. The results are plotted in Figure 8. This plot demonstrates that the number of strongly attached cross-bridges is the same for nfTm and ∆2Tm, and that the number is slightly larger in ∆3Tm. In ∆3Tm, the extra cross-bridges are distributed in the AM and the AM*S states; although there is also some variation in the number of cross bridges in each of the two major force-generating states (AM*DP and AM*D), the sums of the cross-bridges in these states are similar for all three muscle models. These findings lead us to conclude that the force/cross-bridge is less in the ∆3Tm model than in the nfTm and ∆2Tm models. 

### 3.8. Temperature Study

**Figure 9:**
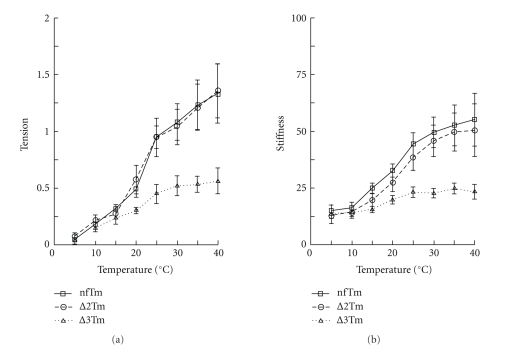
Summary of the temperature effect on isometric tension (a) and stiffness (b). The mean and SEM are shown. The number of experiments is nfTm (*n* = 10), ∆2Tm (*n* = 10), ∆3Tm (*n* = 11). Under the experimental conditions used here (standard activation solution and the constant sarcomere length), the larger temperature sensitivity corresponds to an increased hydrophobic interaction between actin and myosin molecules [24].

Because the positive temperature effect on isometric tension depends on the hydrophobic interaction between actin and myosin [24, 43], and this interaction is hypothesized to change depending on the Tm used to reconstitute the thin filament [13, 14], the temperature study was performed in the standard activating solution while the temperature was varied by 5°C increments, starting at 5°C and going up to 40°C. The results of the tension and stiffness measurements under these conditions are shown in Figures 2 and 9. Figure 2H shows the time course of the tension study, with Figure 2AH representing the nfTm muscle model, Figure 2BH the ∆2Tm model, and Figure 2CH the ∆3Tm model. As these tracings show, isometric tension increased with rising temperature in all muscle models. The increase was similar in the nfTm and ∆2Tm muscle models, but that in ∆3Tm muscle models was significantly lower.

The result of the temperature study is plotted in Figure 9, with respect to both tension (in *A*) and stiffness (in *B*). Figure 9(a) shows that tension increased with rising temperature in all muscle models studied. The plots of the data for the nfTm and ∆2Tm muscle models are indistinguishable, but the plot for the ∆3Tm model is distinctly different, with a slope of about half that of the other two models. The results for stiffness (Figure 9(b)) were similar, with the plots for the nfTm and ∆2Tm muscle model being essentially indistinguishable, but that for the ∆3Tm model being distinctly different and having slope that is less pronounced than those of the two other muscle models. However, the fact that stiffness increased with temperature (1.5–3X) in each muscle model revealed a common feature; specifically, it indicates that more cross-bridges are formed at higher temperatures in all of these models.

### 3.9. Biochemical Analysis of Component Proteins

**Figure 10:**
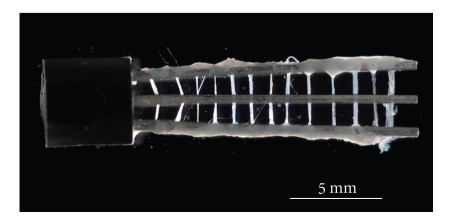
A photomicrograph of a 13 myocardial preparations generated for biochemical analysis.

Fibers used for the mechanical experiments were pooled and used for SDS-PAGE analysis. Because the amount of protein obtained for those experiments was not sufficient for gel electrophoresis, additional preparations of reconstituted fibers were made from 10–15 additional cardiac preparations per mutant (see Methods for details; see also Figure 10).

**Figure 11:**
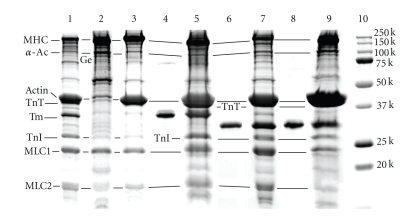
SDS-PAGE of reconstituted myocardium with gradient gel. Lane 1: native myocardium. Lane 2: gelsolin-treated myocardium. Lane 3: actin-filament reconstituted myocardium. Lane 4: purified nfTm. Lane 5: myocardium reconstituted with nfTm and Tn after actin-filament reconstitution. Lane 6: purified ∆2Tm. Lane 7: myocardium reconstituted with ∆2Tm and Tn after actin-filament reconstitution. Lane 8: purified ∆3Tm. Lane 9: myocardium reconstituted with ∆3Tm and Tn after actin-filament reconstitution. Lane 10: molecular weight (MW) markers as labeled. Lanes 1, 4 and 5: same gel. Lanes 6 and 7: same gel. Lanes 8, 9 and 10: same gel. *α*-*A c* = *α*-actinin, Ge = gelsolin. Gelsolin is the band just below the *α*-actinin band in lane 2. ∆2Tm and ∆3Tm run at MW ~30 kD, below the nfTm and native Tm, which run at MW ~34 kD.

Figure 11 shows the results of the SDS-PAGE analysis. Following gelsolin treatment, all thin-filament proteins (actin, Tm, TnT, TnI) were removed, except for the small amount left on the gel (Lane 2; compare to Lane 1, which is native cardiac tissue). The remaining actin was required to nucleate subsequent actin polymerization. The actin filaments were reconstituted (Lane 3), after which Tm and Tn were reconstituted (Lanes 5, 7, 9). TnT is difficult to see because it runs just below the heavy actin band and near 40 kD, as shown in our earlier publications [11, 13]. nfTm, ∆2Tm, and ∆3Tm were all incorporated into reconstituted fibers, as were TnT and TnI (Lanes 5, 7, 9). The staining method used does not visualize TnC. 

Densitometric analysis of the gels demonstrated that the Tm/(actin+TnT) ratio in myocardium reconstituted with recombinant Tms was within the error range of that in native cardiac myocardium (Results not shown). The amount of TnI may have been somewhat lower in the ∆3Tm-reconstituted fibers, but the error of densitometric measurement is large (~20%), so the significance of this is likely minimal.

## 4. Discussion

Tropomyosin is well known to enhance actomyosin interaction. This was first demonstrated by Weber's group [41] who showed that regulatory proteins, troponin and, tropomyosin, in the presence of saturating Ca^2+^, make function the thin filament cooperative, as measured by the actin-activated ATP hydrolysis rate. Eaton [44] demonstrated that HMM cooperatively increases the affinity of Tm for actin; an effect that is consistent with work of Lehrer and Morris [45] who showed a cooperative effect of actin on the activation of the myosin S1 ATPase in the presence of Tm. 

Because the deletion of period 3, but not that of period 2, of Tm's seven repeats impairs the normal function of reconstituted cardiac muscle fibers (Tables 2–5), we infer a specific role for this constitutively expressed period in thin-filament regulation. We previously reported that thin-filament reconstituted with a Tm mutant harboring deletions of both periods 2 and 3 (∆23Tm; deletion of residues 47–123) exhibited impaired tension and stiffness-generating capacity as a consequence not of a reduced number of cross-bridges, but rather due to reduced tension and stiffness per cross-bridge [13]. The temperature dependence of isometric tension and cross-bridge kinetics for this mutant were reduced as well, leading to the suggestion that a hydrophobic interaction contributes to the positive allosteric effect of Tm [14]. The results reported in the current study attribute the impaired function of the ∆23 mutant entirely to the loss of period 3, a constitutively expressed region that is encoded by exon 3. We also show that N-terminal acetylation of Tm is not a requirement for normal contractile function (Figure 3(c)). We thus conclude that cooperative actin-filament activation is influenced by specific internal periods of Tm, but not by N-terminal acetylation, which is primarily a determinant of actin affinity [37, 38]. In contrast, the generic ability of Tm to bind to the actin filament and to stiffen it, thereby stabilizing it against severing proteins such as cofilin, has not been ascribed to specific periods.

The deletion mutant design incorporates the Phillips' [3] proposed actin binding sites based on the heptapeptide repeat of hydrophobic residues that defines the coiled-coil structure. In his analysis, period 2 has 42 residues while period 3 has 35 residues. Previous work showed that ∆3Tm with 35 or 42 residues deleted has similar conformational stability and actin binding properties [15]. For structural considerations we continued our analyses on ∆3Tm with a 35-residue deletion and reported that it impaired the actin affinity, Ca^2+^-dependent activation of the regulated actomyosin ATPase, and cooperative myosin S1-induced binding to actin compared to ∆2Tm [19]. The overall stability of the ∆3Tm mutant is similar to that of the ∆23Tm, in which 77 residues were deleted (= 42 + 35). ∆23Tm binds with higher actin affinity than ∆3Tm [15] but ∆23Tm has altered regulatory function [13, 14] that parallels that of the ∆3Tm mutant. 

Because we observed no noticeable tension development at pCa 7.0 in all 4 Tms tested (Table 1), we conclude that the inhibition of the actomyosin interaction is adequate at pCa 7 in any muscle models we studied.

### 4.1. N-Terminal Acetylation of Tm Does Not Affect Thin-Filament Function in Reconstituted Cardiac Muscle Fibers

The recombinant rat *α*-Tms used to reconstitute the thin filaments in the myocardial strips were expressed in *E . coli*, an organism that lacks the enzyme that carries out post-translational N-terminal acetylation. The acetyl moiety is present in Tms prepared from eukaryotic cells. Because the N-terminal acetylation of Tm is required for the protein's normal affinity for actin, the head-to-tail association among Tm molecules, tropomodulin binding, and the binding of the Tm N-terminal domain to TnT [37, 38, 46, 47], we compared acetylated and unacetylated Tm in our experiments. We found that Ca^2+^ regulation, as assessed by measurements of pCa_50_ and cooperativity (*h*) between neighboring regulatory units and cross-bridges, was essentially the same regardless of acetylation at the N-terminus (Figure 3(c) and Table 2). Furthermore, the kinetic constants of elementary steps of the cross-bridge cycle were not significantly different, except for *k*_−2_ (Table 5). From these results, we conclude that unacetylated Tm has normal regulatory function after it is incorporated into the muscle fiber system, as it does in an in vitro regulated actomyosin system [37]. Similar results have been reported for Tm with an N-terminal Ala-Ser extension [13, 14]. We presume that the binding of our Tm proteins to actin and to the N-terminal domain of TnT “corrects” the structural deficiency of Tm, or that the primary function of N-terminal acetylation is to ensure assembly with the actin filament without being required for subsequent functions.

### 4.2. Comparison to Other Reports

By using ∆23Tm in skeletal myofibrils, a recent report by Siththanandan et al. [48] demonstrated that, at 10°C, pCa_50_ increased by 0.47 units and cooperativity decreased from 2.92 to 1.44, whereas in this report using ∆3Tm in myocardium at 25°C, pCa_50_ increased by 0.23 units and the cooperativity decreased (but insignificantly) from 1.73 to 1.35 (Table 2). Therefore, there is a qualitative agreement between these two reports. Some differences must be based on differences in preparations (skeletal versus cardiac backgrounds), in the reconstitution methods, in the temperature (10°C versus 25°C), and in mutant proteins themselves (AS∆23Tm versus ∆3Tm). In addition, low isometric tension in reconstituted preparations with ∆23Tm (20%) [48] may be a concern. With our methods, isometric tension was 42% in ∆23Tm [13] and 60% in ∆3Tm at 25°C (Table 4). 

For cross-bridge kinetics, Siththanandan et al. [48] measured *k*_act_ and *k*_rel_, but they did not relate these parameters to the kinetic constants of the cross-bridge cycle. If we assume that *k*_rel_ ~ *k*_2_ + *k*_−2_, and  3*k*_act_ ~ (*k*_4_ + *k*_−4_), approximate numerical agreements can be seen among control muscle models, and among mutant (∆23Tm, ∆3Tm) muscle models. These agreements may be fortuitous, however, because we know that the myosin isoform can have a major influence on the cross-bridge kinetics [49, 50], and the ambient temperature difference (10°C  versus 25°C) affects all rate constants significantly, *k*_4_ in particular [24, 43, 51]. Another reason why *k*_act_ is slow may be related to the fact that cross-bridges cycle several times to stretch series elastic elements on activation, hence the rate-limiting step of the cycle dominates *k*_act_ [42]. Furthermore, Siththanandan et al. [48] measured the rate constants of Pi transient at 2 different Pi concentrations, whereas we characterized the force generation step 4 and the Pi release step 5 with 6 different Pi concentrations (Figure 7). Because steps 4 and 5 involve at least 3 independent kinetic parameters (Scheme 1) [35, 52], it would not be possible to deduce mechanisms underlying these steps based on 2 experimental points.

### 4.3. Tm's Allosteric Effects on Actin

Tm's role in the cooperative enhancement of actomyosin interactions has led to the hypothesis that Tm in the presence of Tn and Ca^2+^ induces a positive allosteric effect on actin, and possibly also on myosin [11, 53, 54]. We previously reported that actin-filament reconstituted myocardial preparations undergo greater force development when Tm and Tn are incorporated, demonstrating the positive allosteric effect [11, 13]. We further showed that the force per cross-bridge is about twofold different. Similarly, the inclusion of Tm and Tn in an in vitro motility assay system increased the sliding speed and force per cross-bridge [7–10, 55]. In the case of reconstitution with the ∆23Tm mutant, the force was lower than that produced when the actin filament alone was reconstituted, demonstrating the negative allosteric effect [13, 14]. The similar negative allosteric effect can be seen in ∆3Tm in tension (Table 4, average *T*_*m*_ is less than 1). Based on the results of the current study, we can attribute this negative allosteric effect to the absence of period 3, and suggest that period 3, but not period 2, is essential for the positive allosteric effect that nfTm or acetyl Tm has on the thin filament, and for the enhanced hydrophobic interaction between actin and myosin molecules. These results are consistent with the results of solution assays involving the regulated actomyosin ATPase, in which addition of ∆2Tm was sufficient to rescue regulation [19, 30], whereas the addition of ∆3Tm was not, that is, Ca^2+^ only partially relieved inhibition [19]. While our investigation demonstrates the significance of period 3 for the positive allosteric effect of Tm on actin and excludes period 2 for this effect in the muscle fiber system, it does not rule out the possibility that other periods are also involved in eliciting this positive allosteric effect. Indeed, solution studies have suggested that periods 4 and 5 are involved in this fundamental regulatory function [17, 19, 56], and this possibility needs to be investigated in the muscle fiber system in future studies. It is likely that Tn also plays a role for the allosteric effect and as reported by us [54] as well as others [55].

### 4.4. The Tension per Cross-Bridge, rather than the Number of Force-Generating Cross-Bridges, Is Reduced in the *∆*3Tm Model

Low tension could be the result of a reduced number of force-generating cross-bridges and/or reduced force per cross-bridge. Based on our analysis of the kinetic constants of the elementary steps of the cross-bridge cycle, we estimate that the ∆3Tm model involves the formation of slightly more cross-bridges in the strongly attached force-generating states than in the nfTm or ∆2Tm models (Figure 8). Since the isometric tension is reduced in this model, we calculate the force per cross-bridge in the ∆3Tm model as about one half that in the ∆2Tm, nfTm or acetylated Tm model. The measured isometric tensions in the ∆2Tm and nfTm models are very similar (Table 4); thus we infer that tension/cross-bridge in the ∆2Tm model is unchanged from that in the nfTm model. 

The fact that rigor stiffness is similar among the three Tms tested (Table 4) implies that when the rigor state is established, the connection between actin and myosin becomes similar in each of these models. This observation suggests that the configuration of the AM state in Scheme 1 in the presence of ATP may be different from the rigor-state linkage which exists in the absence of ATP.

### 4.5. Evidence that Tm Period 3 Is Responsible for the Increased Hydrophobic Interaction (Allosteric Effect) between Actin and Myosin Molecules

Many studies on skinned cardiac and skeletal muscle fibers have demonstrated that isometric force increases as the ambient temperature increases [43, 51, 57–65]. Two mechanisms have been proposed to explain this increase: (1) an increase in the number of force-generating cross-bridges as temperature rises, and (2) an increase in the force/cross-bridge as temperature rises. The first mechanism is supported by the fact that the equilibrium constant of the force generation step (*K*_4_) increases with temperature [43, 51, 52, 64, 65]. The second mechanism is supported by the fact that stiffness does not change much with temperature [57, 58, 63, 66], and that there is a structural rearrangement in the cross-bridge conformation (such as axial tilting) based on low angle X-ray diffraction studies [67, 68]. Our finding in the current study, that the stiffness increases as much as 4-fold as temperature increases from 5°C to 40°C (Figure 9(b)), supports the first mechanism as the major cause underlying the temperature effect. While we agree that the cross-bridge conformation may cause a small effect on isometric tension and stiffness, it would be difficult to explain the 4-fold change in stiffness and 13-fold change in tension (Figure 9) based on conformational changes.

The reason for the large temperature effect on *K*_4_ is that the Van der Waals force between hydrophobic (apolar) amino acid residues of actin and myosin has a significant effect on force generation, as shown by a large increase in enthalpy (∆H°), a large increase in entropy (∆S°), and a large decrease in heat capacity (∆C_P_) [43, 51, 64]; reviewed by [24]. When the total number of available cross-bridges is the same, the slope of the temperature-tension plot can be used as an index of the degree of the impact the hydrophobic interaction has on force generation [24]. Our finding that the slope in muscle fibers reconstituted with ∆3Tm instead of nfTm is reduced to half (Figure 9) implies that ∆3Tm leads to an ~50% reduction in the hydrophobic interaction. Our finding that the slope is unchanged when ∆2Tm is used instead of nfTm implies that ∆2Tm does not cause a change in the hydrophobic interaction relative to that due to the presence of nfTm. Thus, the results of the temperature study are consistent with the hypothesis that the hydrophobic interaction between actin and myosin is ~2-fold stronger in fibers reconstituted with nfTm or ∆2Tm than in those reconstituted with ∆3Tm. This observation suggests that period 3 is essential for induction of the positive allosteric effects of Tm on actin, and for an enhanced hydrophobic interaction between actin and myosin molecules. 

According to Holmes et al. [69], there are stereospecific and hydrophobic interactions between residues Pro-529, Met-530, Glu-538, Met-541, Phe-542, Pro-543 of myosin (sequence based on chicken skeletal myosin II), and residues Leu-140, Tyr-143, Ile-341, Ile-345, Leu-349, Phe-352 of actin; similar results had been obtained earlier by Rayment et al. [70]. However, these analyses were carried out in the absence of regulatory proteins. Our results imply that the number of hydrophobic amino acid residues at the actin-myosin interface increases as the regulatory proteins are added and as hypothesized earlier [11, 56]. This is because the allosteric interaction between Tm and actin may modify the actin molecule for better stereoscopic match with the myosin molecule, and that this increased actin-myosin interaction may be further responsible for the increased slope of the temperature-tension plot. Our results indicate that Tm plays a central role in regulating and directing the stereospecific interactions between actin and myosin molecules that lead to optimal force development.

The interaction between Tm and actin is also postulated to include hydrophobic interactions. However, the source of these hydrophobic amino acid residues is not known at the present time. It has long been recognized that the binding of Tm to actin is weaker at low temperature [71], and this is consistent with the idea that the interaction includes a hydrophobic component. The local instability of Tm appears to be essential for actin binding and for the cooperative myosin S1-induced binding of Tm to actin [72, 73]. Periods that are stable in the cold are partially unfolded at physiological temperatures and may confer the flexibility required for Tm to bind to the helical actin filament in this context. Whereas the overall stability of ∆2Tm is similar to that of nfTm, ∆3Tm is less stable, and its lower affinity for actin may reflect a weaker hydrophobic interaction with actin [19]. Because of the weaker interaction between ∆3Tm and actin, this Tm mutant may not be capable of exerting the same allosteric effect on actin that nfTm would. Indeed, this is consistent with our finding that ∆3Tm has a negative allosteric effect. It is important to emphasize that the results presented here were obtained in an organized system in which force can be generated, and that the experiments were carried out at physiological ionic strength. It would be difficult to interpret the results of an experiment if force was not detected. If the experiments were performed in low ionic strength solutions, then the electrostatic interaction among molecular domains would become the major contributing factor, which might invalidate interpretations.

## Figures and Tables

**Table 1:** Resting tension and stiffness. The fibers were relaxed at 0°C in the relaxing solution that contains 40 mM BDM. Tension at pCa7.0 was measured at 25°C as the incremental tension from the relaxed level. All the measured parameters were normalized to *T*_ac_, the active tension of the actin filament reconstituted fibers at 25°C. Compared to the nfTm control, all *P* values are > .05, hence there are no significant differences from nfTm in three parameters measured.

	nfTm (control)	∆2Tm	∆3Tm	Acetyl Tm^†^	Units
Number of expts.	17	18	18	8	
Tension at pCa 7	0.065 ± 0.113	0.109 ± 0.033	0.196 ± 0.047	0.168 ± 0.052	*T*_ac_
Stiffness at pCa 7	0.044 ± 0.015	0.039 ± 0.004	0.040 ± 0.004	0.038 ± 0.011	*T*_ac_
Stiffness at relaxation	0.055 ± 0.022	0.049 ± 0.005	0.044 ± 0.005	0.048 ± 0.015	*T*_ac_

^†^Data obtained together with [13] but were not published.

**Table 2:** Ca^2+^ sensitivity and cooperativity of activation. *P value ≤.05 compared to nfTm; **P value ≤.01 compared to nfTm; else, *P* value >.05 (no significant difference from nfTm). The Hill factor *h* and pCa_50_ (Ca sensitivity) were deduced by fitting the pCa-tension (or pCa-stiffness) data to (1). In all experiments, temperature was 25°C, ionic strength was 200 mM, and pH was adjusted to 7.00. The data were individually fitted to (1), averaged, and tabulated with ±SEM. ^†^Data from [13].

	nfTm (control)	∆2Tm	∆3Tm	Acetyl Tm^†^	Units
Number of expts.	14	15	15	9	
Based on tension					
*h*	1.73 ± 0.21	1.79 ± 0.19	1.35 ± 0.22	1.74 ± 0.23	—
pC_50_	5.28 ± 0.04	5.11 ± 0.07*	5.51 ± 0.08**	5.40 ± 0.08	log_10_
Based on stiffness (*Y*_*∞*_)					
*h*	1.78 ± 0.11	1.86 ± 0.16	1.56 ± 0.22	1.97 ± 0.37	—
pCa_50_	5.33 ± 0.03	5.23 ± 0.05*	5.52 ± 0.07**	5.41 ± 0.09	log_10_

**Table 3:** MgATP^2−^ sensitivity and cooperativity of relaxation. *P value ≤ .05 compared to nfTm; **P value ≤ .01 compare to nfTm; else, *P* value > 0.05. The Hill factor (*g*) and pS_50_ (MgATP^2^- sensitivity) were deduced by fitting the pS-tension (or stiffness) data to (2), where pS = –log_10_(*S*) and *S* = [MgATP]. In all experiments, temperature was 25°C, ionic strength was 200 mM, and pH was adjusted to 7.00. The data were individually fitted to (2), averaged, and tabulated with ± SEM.

	nfTm	∆2Tm	∆3Tm	Units
Number of expts.	5	5	4	
Based on tension				
*g*	7.8 ± 3.1	5.9 ± 4.0	7.1 ± 3.6	—
pS_50_	5.12 ± 0.05	5.00 ± 0.08	4.95 ± 0.06*	log_10_
Based on stiffness (*Y*_*∞*_)				
*g*	2.1 ± 0.5	2.0 ± 0.5	3.0 ± 0.9	—
pS_50_	5.28 ± 0.05	5.22 ± 0.09	4.99 ± 0.07**	log_10_

**Table 4:** Measured parameters of Cross-bridges. *P value ≤ .05 compared to nfTm; **P value ≤.01 compared to nfTm; else, *P* value > .05. Other than rigor stiffness, all parameters were measured in the standard activating solution. *T*_*m*_ is the steady-state active tension. Tension and stiffness were normalized against the actin filament tension (*T*_ac_) in the standard activating solution at 25°C (*T*_ac_ = 9.0 ± 0.9 kPa, ± SEM, *N* = 52). To deduce *f*_min_, 5 frequency points around the minimum |*Y*(*f*)| are fitted to the cubic equation with 4 constants. 2*π*b corresponds to the rate constant of phase 3, and 2*π*c corresponds to that of phase 2 of step analysis [32]. In all experiments, temperature was 25°C, ionic strength 200 mM, and pH 7.00. The rigor stiffness was measured at 100 Hz. The data were individually fitted to respective equations, averaged, and tabulated with ± SEM. ^†^Data based on Lu et al. [13].

	nfTm	∆2Tm	∆3Tm	Acetyl Tm^†^	Units
Number of expts.	15	17	18	9	
Tension (*T*_*m*_)	1.51 ± 0.28	1.65 ± 0.29	0.91 ± 0.08*	1.65 ± 0.24	*T*_ac_
*Y*_*∞*_	103 ± 24	103 ± 14	71 ± 5	89 ± 14	*T*_ac_
*T*_*m*_/*Y*_*∞*_	1.56 ± 0.08	1.58 ± 0.14	1.31 ± 0.10*	1.91 ± 0.10**	%*L*_0_
Rigor stiffness	166 ± 11	201 ± 19*	249 ± 59	159 ± 23	*T*_ac_
2*πf*_min_	8.6 ± 0.4	7.4 ± 0.4	7.2 ± 0.4**	7.6 ± 0.6	s^−1^
2*πb*	6.2 ± 0.4	6.3 ± 0.5	4.7 ± 0.4**	7.1 ± 1.0	s^−1^
2*πc*	28 ± 1	28 ± 2	36 ± 3**	41 ± 4**	s^−1^
*H*	49 ± 11	51 ± 9	34 ± 4	28 ± 6*	*T*_ac_
*B*	56 ± 20	55 ± 13	22 ± 2*	29 ± 6	*T*_ac_
*C*	110 ± 34	107 ± 17	58 ± 3	90 ± 13	*T*_ac_

**Table 5:** Kinetic constants of Scheme 1. *P value ≤.05 compared to nfTm; **P value ≤.01 compared to nfTm; else, *P* value >.05. The kinetic constants (for definitions, see Scheme 1) were measured by the sinusoidal length perturbation method at 25°C, ionic strength 200 mM, and pH 7.00. *K*_2_ = *k*_2_ */k*_−2_ and *K*_4_ = *k*_4_ */ k*_−4_. The data were individually fitted to the respective equations, averaged, and tabulated with ± SEM. The number of experiments is given in parentheses ( ). ^†^Data based on Lu et al. [13].

	nfTm	∆2Tm	∆3Tm	Acetyl Tm^†^	Units
*K*_1_	6.9 ± 1.8 (13)	6.6 ± 1.8 (7)	4.6 ± 2.4 (10)	6.2 ± 1.3 (6)	mM^−1^
*K*_2_	2.3 ± 1.0 (13)	1.7 ± 0.5 (7)	1.0 ± 0.4 (10)	1.08 ± 0.21 (6)	—
*K*_4_	0.44 ± 0.08 (10)	0.61 ± 0.11 (13)	1.52 ± 0.54 (10)*	0.61 ± 0.24 (6)	—
*K*_5_	0.13 ± 0.03 (10)	0.30 ± 0.07 (13)*	0.68 ± 0.21 (10)*	0.11 ± 0.03 (6)	mM^−1^
*k*_2_	15 ± 2 (13)	17 ± 2 (7)	14 ± 3 (10)	22 ± 4 (6)	s^−1^
*k*_−2_	13 ± 2 (13)	14 ± 3 (7)	22 ± 3 (10)**	21 ± 2 (6)**	s^−1^
*k*_4_	3.5 ± 0.3 (10)	2.8 ± 0.3 (13)	2.8 ± 0.4 (10)	5.2 ± 1.1 (6)	s^−1^
*k*_−4_	14.1 ± 5.8 (10)	6.0 ± 1.0 (13)	3.8 ± 1.1 (10)*	14.5 ± 3.4 (6)	s^−1^
